# Supplementary material for: Concerns and adjustments: How the Portuguese population met COVID-19
Source: PLoS One. 2020 Oct 14;15(10):e0240500. doi: 10.1371/journal.pone.0240500 (PMC7556479; doi:10.1371/journal.pone.0240500)
Supplement: S1 Table — (PDF) [file pone.0240500.s001.pdf]

S1 Table Survey questions: Social isolation, and concern

Question	Number	%
**Are you in isolation / with social isolation measures implemented?**		
No isolation measures implemented	4,092	55%
With isolation measures implemented	3,329	45%
No details on the type of isolation	1,503	45%
Isolation determined by Health Authorities	80	2%
Voluntary isolation	1,746	52%
No details on the type of voluntary isolation	845	48%
Never/ almost never leaves home	585	34%
Leaves home with frequency (e.g. for work)	316	18%
**How concerned are you with the COVID-19 pandemic?**		
Not concerned	7	0%
Not very concerned	66	1%
Relatively concerned	1,058	14%
Very concerned	3,002	40%
Extremely concerned	3,315	45%
**How concerned are you with the COVID-19 pandemic impact in the Portuguese economy?**		
Not concerned	31	0%
Not very concerned	113	2%
Relatively concerned	751	10%
Very concerned	2,654	36%
Extremely concerned	3,899	52%

Note: Respondents were given the option not to answer particular questions.
% computed based on the number of answers to each question (excludes respondents who opted not to answer).
7,448 valid answers recorded.
