# Supplementary material for: Concerns and adjustments: How the Portuguese population met COVID-19
Source: PLoS One. 2020 Oct 14;15(10):e0240500. doi: 10.1371/journal.pone.0240500 (PMC7556479; doi:10.1371/journal.pone.0240500)
Supplement: S2 Table — (PDF) [file pone.0240500.s002.pdf]

S2 Table Survey questions: Stockpiling and consumption patterns

Question	Number	%
**Did you stockpile groceries given the COVID-19 pandemic?**		
Yes	2,646	36%
No	4,802	64%
**Where were those groceries purchased?**		
Online	214	8%
Neighbourhood market	173	7%
Supermarket	1,290	49%
Hypermarket	969	37%
**How much have you spent on those groceries?**		
<50 euros	640	25%
51-100 euros	1,038	40%
101-300 euros	764	29%
301-500 euros	123	5%
>501 euros	32	1%
**Have you had difficulties in finding goods in supermarkets (select all applicable)*?**		
No	259	48%
Yes	286	52%
Yes - medicines	34	12%
Yes - hygiene products	122	43%
Yes - food supplies	191	67%
Yes - others	58	20%

Note: Respondents were given the option not to answer particular questions.
% computed based on the number of answers to each question (excludes respondents who opted not to answer).
7,448 valid answers recorded.
* Question only included in wave 2.
