# Supplementary material for: Concerns and adjustments: How the Portuguese population met COVID-19
Source: PLoS One. 2020 Oct 14;15(10):e0240500. doi: 10.1371/journal.pone.0240500 (PMC7556479; doi:10.1371/journal.pone.0240500)
Supplement: S3 Table — (PDF) [file pone.0240500.s003.pdf]

S3 Table Survey questions: Health Services capacity

Question	Number	%
**How do you perceive the quality of the health system since the beginning of the COVID-19 pandemic?**		
I am not able to assess	1,064	54%
Worsened substantially	112	6%
Worsened slightly	196	10%
Remains equal	476	24%
Improved slightly	75	4%
Improved substantially	65	3%
**Were any of your existing appointments changed (select all applicable)?**		
I had no appointment(s) planned	1,105	56%
No, my appointments remained unchanged	91	5%
Yes, my appointments were affected	792	40%
Changed to a remote appointment	49	6%
Doctor appointment cancelled / postponed	602	76%
Surgery cancelled / postponed	30	4%
Medical procedure cancelled / postponed	16	2%
Other appointments cancelled / postponed	176	22%
**Where were your appointments (select all applicable)*?**		
NHS Hospital	99	22%
NHS Primary Care	112	25%
Private sector	256	56%
Others	33	7%
**Do you have any health insurance / health sub-system (select all applicable)?**		
No	467	24%
Yes	1,510	76%
Yes, a health subsystem	520	26%
Yes, health insurance provided by the employer	448	23%
Yes, voluntary health insurance	643	33%

Note: *Type of service not recorded for consultations changed to remote. Respondents were given the option not to answer particular questions.
% computed based on the number of answers to each question (excludes respondents who opted not to answer).
1,988 valid answers recorded.
Questions not included in wave 1.
