# Supplementary material for: Concerns and adjustments: How the Portuguese population met COVID-19
Source: PLoS One. 2020 Oct 14;15(10):e0240500. doi: 10.1371/journal.pone.0240500 (PMC7556479; doi:10.1371/journal.pone.0240500)
Supplement: S4 Table — (PDF) [file pone.0240500.s004.pdf]

S4 Table Survey questions: Economic consequences

Question	Number	%
**Have you felt the impact from COVID-19 in your current financial situation?**		
No impact	672	34%
Yes, positive impact	35	2%
Small impact	9	26%
Moderate impact	18	51%
High impact	8	23%
Very high impact	0	0%
Yes, negative impact	1,281	64%
Small impact	147	11%
Moderate impact	597	47%
High impact	379	30%
Very high impact	158	12%
**Do you expect to feel the impact from COVID-19 in your future financial situation?**		
No impact	292	15%
Yes, positive impact	46	2%
Small impact	13	37%
Moderate impact	22	63%
High impact	7	20%
Very high impact	4	11%
Yes, negative impact	1,650	83%
Small impact	222	17%
Moderate impact	711	56%
High impact	496	39%
Very high impact	221	17%

Note: Respondents were given the option not to answer particular questions.
% computed based on the number of answers to each question (excludes respondents who opted not to answer).
1,988 valid answers recorded.
Questions not included in wave 1.
