# Supplementary material for: Concerns and adjustments: How the Portuguese population met COVID-19
Source: PLoS One. 2020 Oct 14;15(10):e0240500. doi: 10.1371/journal.pone.0240500 (PMC7556479; doi:10.1371/journal.pone.0240500)
Supplement: Attachment — Submitted filename: Response to Reviewers.pdf [file pone.0240500.s007.pdf]

Dear Professor Renuka Sane,

Thank you very much for the helpful comments and for the opportunity to revise our manuscript. We acknowledge the need of revisions and tried to answer to all observations, as described below.

According to your feedback we have carefully revised the wording/ phrasing of the document, as to improve its style. Additionally, we have included a specific mention regarding the non-representative sample both in the abstract and results section – in addition to the references made in the Discussion section.

Please let us know should you have any questions.

Kind regards,

Sara Valente de Almeida

Eduardo Costa

Francisca Vargas Lopes

João Vasco Santos

Pedro Pita Barros
